# The Usefulness of the Ratio of Antigen–Autoantibody Immune Complexes to Their Free Antigens in the Diagnosis of Non-Small Cell Lung Cancer

**DOI:** 10.3390/diagnostics13182999

**Published:** 2023-09-20

**Authors:** Heyjin Kim, Jin Kyung Lee, Ae-Chin Oh, Hye-Ryoun Kim, Young Jun Hong

**Affiliations:** 1 Department of Laboratory Medicine, Korea Cancer Center Hospital, Korea Institute of Radiological and Medical Sciences, Seoul 01812, Republic of Korea; heyjin@kirams.re.kr (H.K.); jklee@kirams.re.kr (J.K.L.);; 2 Division of Pulmonology, Department of Internal Medicine, Korea Cancer Center Hospital, Korea Institute of Radiological and Medical Sciences, Seoul 01812, Republic of Korea; slowly7@kirams.re.kr

**Keywords:** lung cancer, non-small cell lung cancer, autoantibody, biomarker, CYFRA21-1, ProGRP, NGAL, NSE

## Abstract

Autoantibodies against specific lung cancer-associated antigens have been suggested for the performance of lung cancer diagnosis. This study aimed to evaluate the diagnostic performance of the antigen–autoantibody immune complex (AIC) against its free antigens for CYFRA21-1, ProGRP, neutrophil gelatinase-associated lipocalin (NGAL), and neuron-specific enolase (NSE) in non-small cell lung cancer (NSCLC). In total, 85 patients with NSCLC and 120 healthy controls (HCs) were examined using a 9-guanine DNA chip method. The ratios of AICs to their antigens and the combinations of ratios consisting of two to four markers were calculated. The levels of AICs for CYFRA21-1, ProGRP, NGAL, and NSE were higher than those for their free antigens in all participants. The levels of each free antigens distinguished patients with NSCLC from the HCs. The ratios of the AIC to its antigen and seven combinations of two to four ratios were significantly higher in patients with NSCLC than in the HCs. Excellent diagnostic performance was observed for all combination ratios (C4-1), with 85.9% sensitivity and 86.7% specificity at a 3.51 cut-off. Higher sensitivity was observed in the early stages (0–I) and adenocarcinoma than in stages II–IV and other pathological types. Combining all ratios of AICs and their antigens for all four markers was useful when diagnosing NSCLC.

## 1. Introduction

Lung cancer is among the most common and deadliest cancers worldwide. Most lung cancers are classified into two categories: non-small cell lung cancer (NSCLC) and small cell lung cancer (SCLC), with NSCLC accounting for more than 80% of lung cancer cases. Although recent declines in mortality have been reported, likely due to reductions in smoking, advances in early detection, and major advances in the treatment of NSCLC, the 5-year relative survival rate for lung cancer is still dismal at 22% overall [1]. The diagnosis of lung cancer is highly dependent on imaging technologies. Lung cancer screening using low-dose computed tomography (LDCT) has been shown to reduce lung cancer deaths [2]. Therefore, LDCT is a promising method for diagnosing lung cancer. However, it has the major disadvantages of high false-positive rates and costs [2,3]. Diagnosis at early stages (0–I) compared to metastatic stages (III–IV) has great potential to reduce mortality and increase the 5-year survival rate by up to 60% in patients with lung cancer [2,4]. However, only 24% of lung cancers are diagnosed at an early stage because signs and respiratory symptoms do not usually appear until the cancer advances [1]. Several candidates have been reported as cancer-associated biomarkers to detect lung cancer using peripheral blood and targeting different sources, such as circulating tumour cells and genetic, epigenetic, proteomic, and metabolic materials [5,6,7,8,9,10]. Tumour markers, such as cytokeratin 19 fragment (CYFRA21-1), carcinoembryonic antigen, neuron-specific enolase (NSE), squamous cell carcinoma antigen, pro-gastrin-releasing peptide (proGRP), and cancer antigen 125, are currently used in clinical laboratories for the diagnosis and monitoring of lung cancer, but they are underutilised owing to their unsatisfactory diagnostic performance, particularly for lung cancer [11].

Autoantibodies are produced and circulated early in the immune systems of patients after exposure to cancer proteins and have attracted attention as useful markers for the early detection of cancer [12,13]. Moreover, even if the antigen exists at a low concentration, autoantibodies are produced at a relatively high concentration and have a continuous blood concentration and a long half-life owing to limited proteolysis and removal [14]. However, detecting autoantibodies in the blood of patients with cancer has been technically challenging. As well as traditional biomarkers, such as p53, CYFRA21-1, ProGRP, and NSE, several biomarkers associated with lung cancer, such as c-Myc, survivin, HER2, NY-ESO-1, and SOX1, have been used in various diagnostic technologies based on the concept of autoantibodies [4,6,15,16,17]. In addition, their diagnostic performance in lung cancer was improved by combining different biomarkers rather than using a single autoantibody [17,18,19,20,21]. However, the different methods used to detect these materials have insufficient sensitivity and specificity for use as diagnostic sensors in clinical laboratories [17,18,19,20,21,22]. A recent study using the Early-CDT^®^ Lung Kit, which measures seven autoantibodies against p53, SOX2, CAGE, NY-ESO-1, GBU4-5, MAGE A4, and HuD, reported a sensitivity of 21% sensitivity for early stage (I–II) and an overall sensitivity of 33% with a specificity of 88% for lung cancer [23].

A new method that uses a 9-guanine DNA chip to measure the antigen–autoantibody immune complex (AIC) and its antigen based on the ratio of AIC to its free antigen was verified for its efficiency in detecting lung cancer [24,25,26]. Previously, tumour marker-specific autoantibodies were overexpressed in patients with cancer [27]. However, the amount and type of tumour-specific proteins that increase in each individual may differ during cancer development. Therefore, if a specific cut-off is used to discriminate between patients with cancer and healthy individuals, the overlap with healthy individuals will result in a lower specificity, which was also observed for AICs and free antigens separately measured in a previous study using the same method [24,25,26]. Irrespective of the number of tumour-specific proteins present in each individual, the ratio of AIC to its free antigen confirmed its potential as a marker to discriminate between the healthy controls (HCs) and patients with early-stage lung cancer [24,25]. The clinical usefulness of AIC and its free antigen against CYFRA21-1 has been reported in the screening of lung cancers, with 76.0% sensitivity and 87.5% specificity [24]. Using the same method, researchers have attempted to develop immunoassays using several candidate markers to improve the diagnostic efficiency of lung cancer detection. This study investigated possible candidate biomarkers for NSCLC by measuring the AIC and its free antigen for four proteins (CYFRA21-1, ProGRP, neutrophil gelatinase-associated lipocalin [NGAL], and NSE), using a 9-guanine DNA chip to detect stage 0 (carcinoma in situ [CIS])–IV NSCLC.

## 2. Materials and Methods

### 2.1. Study Participants

Clinical samples derived from patients with NSCLC (*n* = 85) and HCs (*n* = 120) were collected and tested in 2019 at the Korea Cancer Central Hospital. We retrospectively reviewed patients’ electronic medical records. All patients with NSCLC were screened using chest radiography and LDCT, followed by a biopsy of individuals with abnormal LDCT findings. Pathologically, the patient was not diagnosed with a subtype of NSCLC and was classified into other types. Two patients with CIS diagnosed with adenocarcinoma in situ were classified as having adenocarcinoma. The two cases of bronchoalveolar carcinomas were reclassified as adenocarcinomas (Table 1). All HCs were screened for lung cancer using chest radiography. Patients with a history of cancer were excluded through a questionnaire administered during the annual health check-up program. Patients with infectious diseases at the time of evaluation were excluded. Blood samples from patients with NSCLC were collected before treatment, such as surgery, chemotherapy, and radiotherapy. K2 ethylenediaminetetraacetic acid (EDTA)-anticoagulated remnant blood specimens were obtained from all participants after a complete blood cell count test. All samples were stored at 4 °C before centrifugation for 10 min at 2000× *g* at 4 °C. Plasma samples were then archived in the biobank at −70 °C. Samples used in this study were obtained from the Korea Institute of Radiological and Medical Sciences (KIRAMS) Radiation Biobank (KRB-2019-I006). We selected eight candidate markers of four proteins (CYFRA21-1, ProGRP, NGAL, and NSE) that have been reported to be useful in the detection of lung cancer in previous studies [28,29,30,31,32,33]. Eight markers were measured in plasma samples from all participants: CYFRA21-1-anti-CYFRA21-1 AIC (CIC), CYFRA21-1 antigen, ProGRP-anti-ProGRP AIC (PrGIC), ProGRP antigen, NGAL-anti-NGAL AIC (NGIC), NGAL antigen, NSE-anti-NSE AIC (NSIC), and NSE. This study was approved by the Institutional Review Board of the Korea Institute of Radiological and Medical Sciences (KIRAMS) (IRB#-2019-07-009-001).

### 2.2. Methods

Based on previous studies, bioconjugates were prepared using six types of syntheses—marker protein-capture antibody (cAb)-DNA, anti-mouse IgG-Cy5, anti-human IgG-Cy5, marker protein-detection Ab (dAb)-fluorescent beads (FB), anti-human-IgG-FB, and anti-human-IgG-FB—and Cy5-DNA for four protein markers, including CYFRA21-1, ProGRP, NGAL, and NSE [24,26]. The lateral flow strip membranes (LFSM) were also prepared to detect the eight markers (CIC, CYFRA21-1, PrGIC, ProGRP, NGIC, NGAL, NSIC, and NSE). The LFSM was manufactured based on the 9-guanine DNA membranes. The 9-guanine DNA membranes were lined with 18 pmol/L solutions of oligonucleotide Probe 1–Probe 8 appended with nine consecutive guanines (9G) corresponding to the test line and the hybridisation control line and allowed to immobilise. After immobilisation, the membranes were soaked in a blocking solution and dried. 

The measurements of CIC, CYFRA21-1, PrGIC, ProGRP, NGIC, NGAL, NSIC, and NSE in plasma samples derived from HCs and patients with NSCLC were performed using a sandwich immunoassay DNA-guided method based on a 9-guanine DNA chip (Biometrix Technology Inc., Chuncheon, Republic of Korea). The 9-guanine DNA chip quantified the plasma levels of all markers in 30 min at 25 °C: for free antigen (or AIC) detection, a 20-microlitre plasma sample was incubated with 100 μL of the solution containing a marker protein-cAb-DNA conjugate (10 fmol/mL), a marker protein-dAb-FB conjugate (or anti-hum-IgG-FB conjugate for AIC detection) (0.07 fmol/mL), and FB-DNA (10 fmol/mL) in an e-tube and incubated at 25 °C for 10 min in a thermo-controller. After the incubation step, 60 μL of reaction buffer containing FB-DNA complementary to the DNA immobilised on the hybridisation control line was added to the reaction tube. The reaction mixture was then loaded onto the LFSM and hybridised for 10 min at 25 °C. Highly specific DNA-DNA hybridisation allowed the capture of the marker protein-dAb-FB-a marker protein-cAb-DNA for free antigen detection or the anti-human-IgG-FB-AIC-a marker protein-cAb-DNA for AIC detection, bimolecular complexes, and Cy5-DNA on the test line and hybridisation control line, respectively. The unbound biomolecular complexes were then washed away at 25 °C in a washing step that required 10 min to add 170 mL of the washing solution (0.1% SDS in 4 × SSC, pH 7.4). After washing, the bound materials of each marker were detected and quantified by scanning the LFSM using a BMT Membrane Reader^TM^ (Biometrix Technology Inc., Chuncheon, Republic of Korea). The fluorescence intensity was expressed in arbitrary units according to validated standard curves [24,26].

The levels of CIC, CYFRA21-1, PrGIC, ProGRP, NGIC, NGAL, NSIC, and NSE in plasma samples from the HCs and patients with NSCLC were recorded. These levels were calculated using the ratios of CIC to CYFRA21-1, PrGIC to ProGRP, NGIC to NGAL, and NSIC to NSE. In particular, CYFRA21-1 was required in the combination equations because it is the leading marker in the diagnosis of NSCLC with the highest sensitivity, using either the same or different methods [24,34]. Finally, the combination ratios for two to four markers, including CIC/CYFRA21-1, were determined according to Equations (1)–(7) and used to discriminate between HCs and patients with NSCLC.

C2-1 = [(CIC/CYFRA21-1) × (PrGIC/ProGRP)](1)

C2-2 = [(CIC/CYFRA21-1) × (NGIC/NGAL)](2)

C2-3 = [(CIC/CYFRA21-1) × (NSIC/NSE)](3)

C3-1 = [(CIC/CYFRA21-1) × (PrGIC/ProGRP) × (NGIC/NGAL)](4)

C3-2 = [(CIC/CYFRA21-1) × (PrGIC/ProGRP) × (NGIC/NGAL)](5)

C3-3 = [(CIC/CYFRA21-1) × (NGIC/NGAL) × (NSIC/NSE)](6)

C4-1 = [(CIC/CYFRA21-1) × (PrGIC/ProGRP) × (NGIC/NGAL) × (NSIC/NSE)](7)

Sensitivity and specificity were determined using cut-off levels to distinguish between the HCs and patients with NSCLCs. Ultimately, the cut-off levels were first aimed at the area under the curve (AUC), before being aimed at the minimal difference between sensitivity and specificity [35]. 

### 2.3. Statistical Analysis

The data were not normally distributed. Continuous data were expressed as medians with interquartile ranges (IQR). Categorical data were presented as counts and percentages. The difference between the HCs and patients with NSCLC was calculated using the Mann–Whitney U test. For all analyses, two-sided *p* values < 0.05 were considered to be statistically significant. The receiver operating characteristic (ROC) curve was used to assess the overall diagnostic performance, and Delong’s method was used to compare the ROC curves. The optimal cut-off point in the coordinates of the ROC curve was determined using the closest top-left method. The sensitivity, specificity, accuracy, positive predictive value (PPV), and negative predictive value (NPV) were calculated at 95% confidence intervals (CI). Statistical analyses were performed using SPSS software version 22 (IBM Corp., Armonk, NY, USA) and Rex-Pro version 3.6.1.0 (RexSoft Inc., Seoul, Republic of Korea). 

## 3. Results

### 3.1. The Levels of AICs and Their Antigens for CYFRA21-1, ProGRP, NGAL, and NSE

The characteristics of the participants in the NSCLC and the HC groups are shown in Table 1. The levels of the AICs (CIC, PrGIC, NGIC, and NSIC) were significantly higher than those of their free antigens (CYFRA21-1, ProGRP, NGAL, and NSE) in all participants (all *p* < 0.0001), HCs (*p* < 0.0001, *p* = 0.0027, *p* = 0.0198, and *p* < 0.0001, respectively) and patients with NSCLC (all *p* < 0.0001) (Figure S1). 

### 3.2. Diagnostic Performance

**Figure 1:**
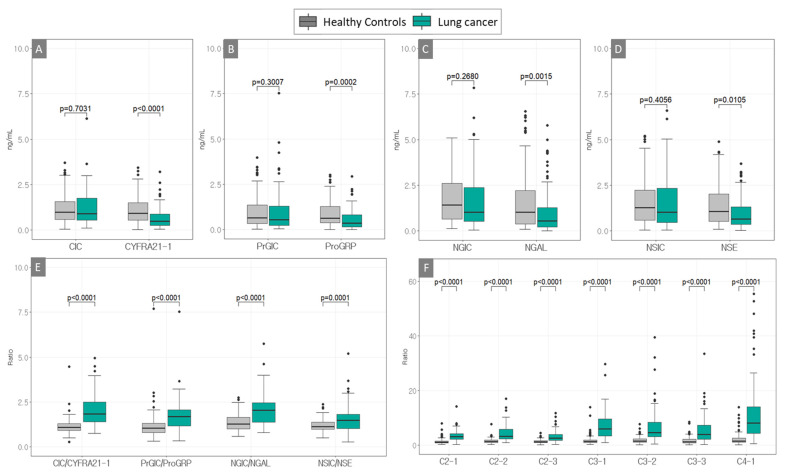
Boxplots used to distinguish patients with lung cancer (non-small cell lung cancer) and the healthy controls using four protein markers, including antigen–autoantibody immune complexes and their free antigens (**A**–**D**), their ratios (**E**) and combination ratios of two to four markers (**F**), including (**A**) CIC and CYFRA21-1, (**B**) ProGIC and ProGRP, (**C**) NGIC and NGAL, and (**D**) NSIC and NSE. Combinations of the ratios (**E**) CIC/CYFRA21-1, PrGIC/ProGRP, NGIC/NGAL, and NSIC/NSE and (**F**) C2-1 (CIC/CYFRA21-1 × PrGIC/ProGRP), C2-2 (CIC/CYFRA21-1 × NGIC/NGAL), C2-3 (CIC/CYFRA21-1 × NSIC/NSE), C3-1 (CIC/CYFRA21-1 × PrGIC/ProGRP × NGIC/NGAL), C3-2 (CIC/CYFRA21-1 × NGIC/NGAL × NSIC/NSE), C3-3 (CIC/CYFRA21-1 × PrGIC/ProGRP × NSIC/NSE), and C4-1 (CIC/CYFRA21-1 × PrGIC/ProGRP × NGIC/NGAL × NSIC/NSE) (patients with non-small cell lung cancer, *n* = 85; healthy controls, *n* = 120).

**Figure 2:**
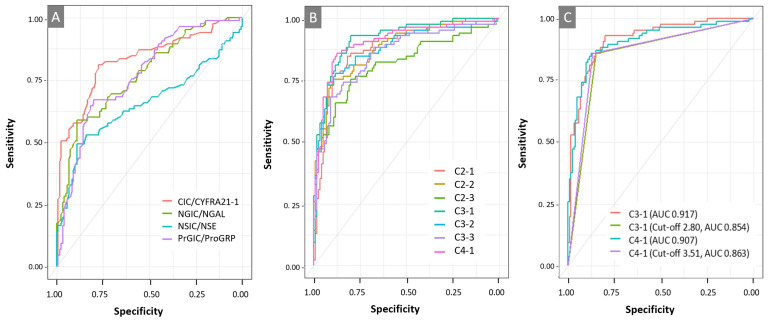
Receiver operating characteristic (ROC) curves: (**A**) ROC curve analysis with the ratios of autoantibody immune complexes to their free antigens (CIC/CYFRA21-1, PrGIC/ProGRP, NGIC, NGAL, NSIC, and NSE); (**B**) ROC curve analysis comparing seven combinations comprising two to four markers, including CIC/CYFRA21-1 (C2-1, C2-2, C2-3, C3-1, C3-2, C3-3, and C4-1); (**C**) comparison of ROC curves for C3-1 and C4-1. Abbreviations: CIC, CYFRA21-1-Anti-CYFRA21-1 autoantibody immune complex; PrGIC, ProGRP-Anti-ProGRP autoantibody immune complex; NGIC, NGAL-Anti-NGAL autoantibody immune complex; NSIC, NSE-Anti-NSE autoantibody immune complex.

The levels of each free antigen more effectively distinguished patients with NSCLC from the HCs than the AIC, and their levels in patients with NSCLC were significantly lower than those in HCs (Figure 1A–D). The ratios of CIC to CYFRA21-1, PrGIC to ProGRP, NGIC to NGAL, and NSIC to NSE were significantly higher in patients with NSCLC than in HCs (Figure 1E). Additionally, the combination ratios (C2-1, C2-2, C2-3, C3-1, C3-2, C3-3, and C4-1) highly discriminated between patients with NSCLC and the HCs (all *p* < 0.0001) (Figure 1F and Table S1). The diagnostic performances of the eight single markers (CIC, CYFRA21-1, PrGIC, ProGRP, NGIC, NGAL, NSIC, and NSE); the ratios of CIC/CYFRA21-1, PrGIC/ProGRP, NGIC/NGAL, and NSIC/NSE; and the seven combinations were compared using ROC analyses (Figure 2 and Table S2). Good diagnostic performance was confirmed based on CIC/CYFRA21-1 among all ratios from the other ratios for a protein, and better diagnostic performance was achieved using combinations of C3-1, C4-1, C2-1, C3-2, C2-2, C3-3, and C2-3 in the order of the large AUC of the ROC (Table S2). Excellent diagnostic performance after applying the optimal cut-off point was obtained using the combinations of C4-1 and C3-1 at the diagnostic cut-offs of 3.51 and 2.80, respectively, which had 85.9% and 85.9% sensitivity and 86.7% and 85% specificity with 0.863 and 0.854 AUC, respectively, in terms of discriminating patients with NSCLC from the HCs (Table 2). However, there was no significant difference between the two ROC curves (*p* = 0.4063) (Figure 2C). The sensitivity and specificity of the C4-1 combination were analysed according to subgroup stages and pathological diagnoses. Higher sensitivity was observed in the very early stages (0–I) of localised tumours than in stages II–IV, with an 86.7% specificity observed for C4-1 (Table 3 and Table S3). Pathological diagnoses were better distinguished in patients with NSCLCs, with the rate of distinguishment being 89.7% for adenocarcinoma and 81.6% for squamous cell carcinoma at 86.7% specificity for C4-1 (Table 4 and Table S4). 

## 4. Discussion

Early screening reduces lung cancer-related deaths by approximately 80% [4]. Several issues related to cost-effectiveness and overdiagnosis due to high false-positive rates have been raised regarding LDCT-dependent diagnostic tools used in lung cancer screening. After confirming that autoantibodies were produced during the early stages of tumorigenesis, their use as diagnostic markers was suggested [36,37]. Studies of autoantibody biomarkers of tumour-associated antigens have been conducted using various methods. However, methods that use blood samples have not been widely adopted in clinical practice because of their low sensitivity and specificity. Moreover, the technical issues involved in measuring autoantibodies can be challenging. 

A recent method using a 9-guanine DNA chip with high affinity to AIC and its free antigen proved to have good clinical applicability [24,25,26]. This study aimed to explore biomarkers used in the diagnosis of NSCLC using a combination of the ratios of AIC to its free antigen for four well-known lung cancer-associated protein markers, including CYFRA21-1, which is one of the most effective discriminators of NSCLC [28,29,30]. Levels of CYFRA21-1 above 10 ng/mL are thought to indicate malignant lung tumours or primary lung cancer, but CYFRA21-1 is released by all lung cancers, regardless of histological type [34]. NGAL is a secreted protein that controls cell proliferation and survival, and its alteration is associated with various malignant tumours, including lung cancer [32,38]. NGAL overexpression is associated with lung adenocarcinoma progression [39]. ProGRP and NSE are diagnostic biomarkers of SCLC with various sensitivities ranging from 47 to 80% [21,32,33,34]. Furthermore, a recent study reported excellent sensitivity and specificity (94.8% and 100%, respectively) for ProGRP in lung neuroendocrine neoplasms [40]. ProGRP and NSE are mainly released by lung cancers, while CYFRA21-1 and NGAL are released by various solid cancers [34]. Much lower levels of free antigens than AICs for the four protein biomarkers were identified in patients with NSCLC, a result that is consistent with those of a previous study [41]. However, no significant difference in the AIC levels was observed between patients with NSCLC and the HCs. This finding is inconsistent with previous studies using different methods that found few tumour-associated autoantibodies in healthy individuals, even in those at a high risk of developing lung cancer [11,42]. However, our results are consistent with those of previous studies using the same method; the ratios of antigen-AICs to their free antigens were more useful than every single marker (AICs and free antigens) in patients with NSCLC [25,26]. Although relatively low sensitivity of AIC/free antigen was observed for ProGRP (67.1%) and NSE (63.5%), a higher sensitivity was observed for the combination of the four fractions (C4-1) for CYFRA21-1, ProGRP, NGAL, and NSE. Although all patients were diagnosed with NSCLC, it was detected at stages 0/I–IV with 85.9% sensitivity and 86.7% specificity compared to other combinations of ratios or a single ratio (Table 3). The diagnostic performance was more pronounced in early-stage lung cancer (stage 0/I) or localised tumours, having over 90% sensitivity and 86.7% specificity, reflecting the characteristics of tumour-associated autoantibodies. 

This study had several limitations owing to the small number of retrospective cohort studies included. The levels of AIC, free antigens, and their ratios did not allow us to assess the differences in sex and age. However, no differences in autoantibodies based on age, sex, or race were reported in a previous study that used a large cohort [39]. A small number of participants, especially those with stage 0, II, or IV NSCLC, were included in this study. Although a significant difference after applying C4-1 was observed between patients with stage II NSCLC and the HCs, there was lower sensitivity in stage II than in the other stages (Table 3). Additionally, lower sensitivity was observed in the regional tumour state, comprising stages IIb and III. Furthermore, 12 of the 16 stage II patients did not have adenocarcinoma (squamous cell carcinoma, 10; other NSCLCs, 2) and showed lower sensitivity than for adenocarcinoma (Table 4). Pathologically, the diagnostic efficiency was higher for adenocarcinoma than for squamous cell carcinoma or other NSCLCs. 

Consistent with previous studies of CYFRA21-1 in lung cancer, squamous cell carcinoma was well discriminated by the single ratio of CIC/CYFRA21-1, having higher sensitivity than other pathological types (adenocarcinoma and other NSCLCs) (84.2% vs. 78.9%, 77.8%, respectively) (Table S5). However, in squamous cell carcinoma, the AIC/free antigen ratio for proteins, including NGAL and NSE, was less sensitive than or equal to (ProGRP) other tissue types. Therefore, a low sensitivity may be observed in stages with a high proportion of squamous cell carcinomas. These results can also be considered to represent a decrease in the ratio at some intermediate stage (II–III) due to a simultaneous increase in autoantibodies and tumour antigens. Alternatively, this outcome could be related to the increasing inaccuracy of staging in higher stages, which is expected to have lower sensitivity than in early stages [43]. Secondly, clinical information related to potential risk factors of lung cancer, such as smoking history or occupations related to carcinogenic exposure of participants, was not provided [1]. This information may help to validate the currently recommended sensitivities used to identify lung cancer markers. Finally, this study did not evaluate samples with SCLC or benign states, such as lung nodules or infective lung diseases, which could be difficult to diagnose using LDCT. Further research using wider cohorts would help to prove their clinical efficiency in discriminating against lung cancer. It is also worth investigating its utility as a biomarker for the post-treatment monitoring of patients with lung cancer.

## 5. Conclusions

In our study, the ratio of AICs to tumour-associated free antigens was more sensitive in terms of distinguishing patients with NSCLC than measuring individual proteins (AICs and free antigens) using the 9-guanine DNA chip method, which has a high affinity for autoantibodies and antigens present at very low concentrations. Moreover, a combination of all four AIC ratios to and free antigens for CYFRA21-1, ProGRP, NGAL, and NSE discriminated between patients with NSCLC and the HCs, with excellent diagnostic performance. By complementing the imaging-based diagnosis of lung cancer, this test could help to minimise the risk of lung cancer by screening at-risk populations and diagnosing NSCLC at an early stage. This method also has great potential to diagnose NSCLC with clinical efficiency in terms of the accessibility and cost-effectiveness of liquid biopsy.

## Figures and Tables

**Table 1:** Characteristics of the study’s participants (*n* = 205).

Characteristic	Patients with NSCLC (*n* = 85)	Healthy Controls (*n* = 120)
Age, years (median, range)	66 (39–82)	42 (25–66)
Male gender, *n* (%)	70 (82.35%)	60 (50%)
Stage		-
-CIS (0)	2 (2.35%)	
-I	39 (45.88%)	
-II	16 (18.82%)	
-III	24 (28.24%)	
-IV	4 (4.71%)	
Pathologic diagnosis		
-Adenocarcinoma	39 (45.88%)	
-Squamous cell carcinoma	38 (44.71%)	
-Other types	8 (9.41%)	
Pleomorphic carcinoma	4 (4.71%)	
Adenosquamous carcinoma	1 (1.18%)	
High-grade mucoepidermoid carcinoma	1 (1.18%)	
Mucinous carcinoma	1 (1.18%)	
Non-small cell carcinoma *	1 (1.18%)	

Abbreviations: NSCLC, non-small cell lung cancer; CIS, carcinoma in situ. * This case was not determined with a subtype.

**Table 2:** Diagnostic efficiency in terms of sensitivity, specificity, accuracy, PPV, and NPV of the ratios comprising four protein biomarkers and their combinations from the ratios used to distinguish patients with NSCLC (*n* = 85) and the healthy controls (*n* = 120).

Variable	Sensitivity (95% CI)	Specificity (95% CI)	Accuracy (95% CI)	PPV (95% CI)	NPV (95% CI)
CIC/CYFRA21-1	81.2 (71.8–88.8)	77.5 (69.0–84.6)	79.0 (72.8–84.4)	71.9 (61.8–80.6)	85.3 (77.3–91.4)
PrGIC/ProGRP	67.1 (56.0–76.9)	79.2 (70.8–86.0)	74.2 (67.6–80.0)	69.5 (58.4–68.8)	77.2 (68.8–84.3)
NGIC/NGAL	69.4 (58.5–79.0)	70.8 (61.8–78.8)	70.2 (63.5–76.4)	62.8 (52.2–72.5)	76.6 (67.6–84.1)
NSIC/NSE	63.5 (52.4–73.7)	60.8 (51.5–69.6)	62.0 (54.9–68.6)	53.5 (43.3–63.5)	70.2 (60.4–78.8)
C2-1	85.9 (76.6–92.5)	80.0 (71.7–86.8)	82.4 (76.5–87.4)	75.3 (65.5–83.5)	88.9 (81.4–94.1)
C2-2	81.9 (71.2–88.8)	78.3 (69.9–85.3)	79.5 (73.3–84.8)	72.6 (62.5–81.3)	85.5 (77.5–91.5)
C2-3	76.5 (66.0–85.0)	76.7 (68.1–83.9)	76.6 (70.2–82.2)	69.9 (59.5–79.0)	82.1 (73.8–88.7)
C3-1	85.9 (76.6–92.5)	85.0 (77.3–90.9)	85.4 (79.8–89.9)	80.2 (70.6–87.8)	89.5 (82.3–94.4)
C3-2	84.7 (75.3–91.6)	77.5 (69.0–84.6)	80.5 (74.4–85.7)	72.7 (62.9–81.2)	87.7 (80.0–93.3)
C3-3	75.2 (64.8–84.0)	76.7 (68.1–83.9)	76.1 (69.7–81.8)	69.6 (59.1–78.7)	81.4 (73.0–88.1)
C4-1	85.9 (76.6–92.5)	86.7 (79.3–92.2)	86.3 (80.9–90.7)	82.0 (72.5–89.4)	89.7 (82.6–94.5)

Abbreviations: CIC, CYFRA21-1-Anti-CYFRA21-1 autoantibody immune complex; PrGIC, ProGRP-Anti-ProGRP autoantibody immune complex; NGIC, NGAL-Anti-NGAL autoantibody immune complex; NSIC, NSE-Anti-NSE autoantibody immune complex; PPV, positive predictive value; NPV, negative predictive value; CI, confidence interval.

**Table 3:** The diagnostic efficiency of the C4-1 according to stages in patients with NSCLC.

Stage (Number)	Sensitivity (95% CI)	Specificity (95% CI)	PPV (95% CI)	NPV (95% CI)
CIS (*n* = 2)	100 (19.8–100)	86.7 (79.3–92.2)	11.1 (1.9–36.1)	100 (95.6–100)
Stage I (*n* = 39)	89.7 (74.8–96.7)		68.6 (54.0–80.5)	96.3 (90.2–98.8)
Stage II (*n* = 16)	62.5 (35.9–83.7)		38.5 (20.9–59.3)	94.5 (88.0–97.8)
Stage III (*n* = 24)	91.7 (71.5–98.5)		57.9 (40.9–73.2)	98.1 (92.7–99.7)
Stage IV (*n* = 4)	100 (39.6–100)		20.0 (6.6–44.3)	100 (55.7–93.4)
Very early stage CIS (0)–I (*n* = 41)	90.2 (75.9–96.8)	86.7 (79.3–92.2)	69.8 (55.5–81.3)	96.3 (90.2–98.8)
Stage II–IV (*n* = 44)	81.8 (64.2–89.7)		69.2 (54.7–80.9)	92.9 (86.0–96.6)

Abbreviations: CIS, carcinoma in situ; PPV, positive predictive value; NPV, negative predictive value; CI, confidence interval.

**Table 4:** The diagnostic efficiency of C4-1 according to pathologic diagnosis in patients with NSCLC.

Type (Number)	Sensitivity (95% CI)	Specificity (95% CI)	PPV (95% CI)	NPV (95% CI)
NSCLC (*n* = 85)	85.9 (76.6–92.5)	86.7 (79.3–92.2)	82.0 (72.5–89.4)	89.7 (82.6–94.5)
Adenocarcinoma (*n* = 39, 25/4/9/1 *)	89.7 (74.8–96.6)		68.6 (54.0–80.5)	96.3 (90.2–98.8)
Squamous cell carcinoma (*n* = 38, 14/10/12/2 *)	81.6 (65.1–91.7)		66.0 (50.6–78.7)	93.7 (87.0–97.2)
Other NSCLCs (*n* = 8, 2/2/3/1 *)	87.5 (46.7–99.3)		30.4 (14.1–53.0)	99.0 (94.0–100.0)

* Total number of each pathologic type and the number divided into patients with stages 0-I/II/III/IV. Abbreviations: PPV, positive predictive value; NPV, negative predictive value; CI, confidence interval.

## Data Availability

Data sharing not applicable.

## Supplementary Materials

The following supporting information can be downloaded via the following link: https://www.mdpi.com/article/10.3390/diagnostics13182999/s1, Figure S1: The levels of AICs and their antigens for CYFRA21-1, ProGRP, NGAL, and NSE in all participants, healthy controls, and patients with lung cancer. Table S1: Levels of antigen–autoantibody immune complexes, free antigens, their ratios, and combinations from two to four ratios for four proteins; Table S2: Area under the curve (AUC) for each marker; Table S3: The diagnostic efficiency of the C4-1 including false positive and false negative rates according to stage; Table S4: The diagnostic efficiency of C4-1 including false positive and false negative rates according to pathological diagnosis; Table S5: The diagnostic efficiency of each ratio for the four proteins according to pathological diagnosis.

## References

[1] American Cancer Society (ACS) (2022). Cancer Facts & Figures. https://www.cancer.org/research/cancer-facts-statistics/all-cancer-facts-figures/cancer-facts-figures-2022.html.

[2] Jonas D.E., Reuland D.S., Reddy S.M., Nagle M., Clark S.D., Weber R.P., Enyioha C., Malo T.L., Brenner A.T., Armstrong C. (2021). Screening for lung cancer with low-dose computed tomography: Updated evidence report and systematic review for the US Preventive Services Task Force. JAMA.

[3] Cui J.W., Li W., Han F.J., Liu Y.D. (2015). Screening for lung cancer using low-dose computed tomography: Concerns about the application in low-risk individuals. Transl. Lung Cancer Res..

[4] Henschke C.I., Yankelevitz D.F., Libby D.M., Pasmantier M.W., Smith J.P., Miettinen O.S., International Early Lung Cancer Action Program Investigators (2006). Survival of patients with stage I lung cancer detected on CT screening. N. Engl. J. Med..

[5] Goebel C., Louden C.L., Mckenna R., Onugha O., Wachtel A., Long T. (2020). Blood test shows high accuracy in detecting stage I non-small cell lung cancer. BMC Cancer.

[6] Zamay T.N., Zamay G.S., Kolovskaya O.S., Zukov R.A., Petrova M.M., Gargaun A., Berezovski M.V., Kichkailo A.S. (2017). Current and prospective protein biomarkers of lung cancer. Cancers.

[7] Pennell N.A., Arcila M.E., Gandara D.R., West H. (2019). Biomarker testing for patients with advanced non-small cell lung cancer: Real-world issues and tough choices. Am. Soc. Clin. Oncol. Educ. Book.

[8] Duruisseaux M., Esteller M. (2018). Lung cancer epigenetics: From knowledge to applications. Semin. Cancer Biol..

[9] Qi S.A., Wu Q., Chen Z., Zhang W., Zhou Y., Mao K., Li J., Li Y., Chen J., Huang Y. (2021). High-resolution metabolomic biomarkers for lung cancer diagnosis and prognosis. Sci. Rep..

[10] Rossi E., Aieta M., Tartarone A., Pezzuto A., Facchinetti A., Santini D., Ulivi P., Ludovini V., Possidente L., Fiduccia P. (2021). A fully automated assay to detect the expression of pan-cytokeratins and of EML4-ALK fusion protein in circulating tumour cells (CTCs) predicts outcome of non-small cell lung cancer (NSCLC) patients. Transl. Lung Cancer Res..

[11] Qin J., Zeng N., Yang T., Wan C., Chen L., Shen Y., Wen F. (2018). Diagnostic value of autoantibodies in lung cancer: A systematic review and meta-analysis. Cell Physiol. Biochem..

[12] Dunn G.P., Bruce A.T., Ikeda H., Old L.J., Schreiber R.D. (2002). Cancer immunoediting: From immunosurveillance to tumor escape. Nat. Immunol..

[13] Solassol J., Maudelonde T., Mange A., Pujol J.L. (2011). Clinical relevance of autoantibody detection in lung cancer. J. Thorac. Oncol..

[14] Pedersen J.W., Wandall H.H. (2011). Autoantibodies as biomarkers in cancer. Lab. Med..

[15] Chapman C.J., Murray A., McElveen J.E., Sahin U., Luxemburger U., Türeci O., Wiewrodt R., Barnes A.C., Robertson J.F. (2008). Autoantibodies in lung cancer: Possibilities for early detection and subsequent cure. Thorax.

[16] Rohayem J., Diestelkoetter P., Weigle B., Oehmichen A., Schmitz M., Mehlhorn J., Conrad K., Rieber E.P. (2000). Antibody to the tumor-associated inhibitor of apoptosis protein survivin in cancer patients. Cancer Res..

[17] Yang B., Li X., Ren T., Yin Y. (2019). Autoantibodies as diagnostic biomarkers for lung cancer: A systematic review. Cell Death Discov..

[18] Boyle P., Chapman C.J., Holdenrieder S., Murray A., Robertson C., Wood W.C., Maddison P., Healey G., Fairley G.H., Barnes A.C. (2011). Clinical validation of an autoantibody test for lung cancer. Ann. Oncol..

[19] Zhang J.Y., Casiano C.A., Peng X.X., Koziol J.A., Chan E.K., Tan E.M. (2003). Enhancement of antibody detection in cancer using panel of recombinant tumor-associated antigens. Cancer Epidemiol. Biomark. Prev..

[20] Zhong L., Coe S.P., Stromberg A.J., Khattar N.H., Jett J.R., Hirschowitz E.A. (2006). Profiling tumor-associated antibodies for early detection of non-small cell lung cancer. J. Thorac. Oncol..

[21] Broodman I., Lindemans J., van Sten J., Bischoff R., Luider T. (2017). Serum protein markers for the early detection of lung cancer: A focus on autoantibodies. J. Proteome Res..

[22] Dai N., Cao X.J., Li M.X., Qing Y., Liao L., Lu X.F., Zhang S.H., Li Z., Yang Y.X., Wang D. (2013). Serum APE1 autoantibodies: A novel potential tumor marker and predictor of chemotherapeutic efficacy in non-small cell lung cancer. PLoS ONE.

[23] Borg M., Wen S.W.C., Nederby L., Hansen T.F., Jakobsen A., Andersen R.F., Weinreich U.M., Hilberg O. (2021). Performance of the EarlyCDT^®^ Lung test in detection of lung cancer and pulmonary metastases in a high-risk cohort. Lung Cancer.

[24] Song K.S., Nimse S.B., Warkad S.D., Oh A.C., Kim T., Hong Y.J. (2019). Quantification of CYFRA 21–1 and a CYFRA 21–1–anti-CYFRA 21–1 autoantibody immune complex for detection of early stage lung cancer. Chem. Commun..

[25] Choe W., Chae J.D., Lee B.H., Kim S.H., Park S.Y., Nimse S.B., Kim J., Warkad S.D., Song K.S., Oh A.C. (2020). 9G TestTM cancer/lung: A desirable companion to LDCT for lung cancer screening. Cancers.

[26] Song K.S., Nimse S.B., Warkad S.D., Kim J.H., Kim H.J., Kim T. (2022). Detection and quantification of Tp53 and p53-anti-p53 autoantibody immune complex: Promising biomarkers in early stage lung cancer diagnosis. Biosensors.

[27] Zaenker P., Gray E.S., Ziman M.R. (2016). Autoantibody production in cancer—The humoral immune response toward autologous antigens in cancer patients. Autoimmun. Rev..

[28] Pujol J.L., Molinier O., Ebert W., Daurès J.P., Barlesi F., Buccheri G., Paesmans M., Quoix E., Moro-Sibilot D., Szturmowicz M. (2004). CYFRA 21–1 is a prognostic determinant in non-small-cell lung cancer: Results of a meta-analysis in 2063 patients. Br. J. Cancer.

[29] Karnak D., Ulubay G., Kayacan O., Beder S., Ibis E., Oflaz G. (2001). Evaluation of Cyfra 21–1: A potential tumor marker for non-small cell lung carcinomas. Lung.

[30] Wieskopf B., Demangeat C., Purohit A., Stenger R., Gries P., Kreisman H., Quoix E. (1995). Cyfra 21–1 as a biologic marker of non-small cell lung cancer. Evaluation of sensitivity, specificity, and prognostic role. Chest.

[31] Crescenzi E., Leonardi A., Pacifico F. (2021). NGAL as a potential target in tumor microenvironment. Int. J. Mol. Sci..

[32] Shibayama T., Ueoka H., Nishii K., Kiura K., Tabata M., Miyatake K., Kitajima T., Harada M. (2001). Complementary roles of pro-gastrin-releasing peptide (ProGRP) and neuron specific enolase (NSE) in diagnosis and prognosis of small-cell lung cancer (SCLC). Lung Cancer.

[33] Molina R., Auge J.M., Filella X., Viñolas N., Alicarte J., Domingo J.M., Ballesta A.M. (2005). Pro-gastrin-releasing peptide (proGRP) in patients with benign and malignant diseases: Comparison with CEA, SCC, CYFRA 21–1 and NSE in patients with lung cancer. Anticancer Res..

[34] Molina R., Holdenrieder S., Auge J.M., Schalhorn A., Hatz R., Stieber P. (2010). Diagnostic relevance of circulating biomarkers in patients with lung cancer. Cancer Biomark..

[35] Unal I. (2017). Defining an optimal cut-point value in ROC analysis: An alternative approach. Comput. Math. Methods Med..

[36] Tan E.M., Zhang J. (2008). Autoantibodies to tumor-associated antigens: Reporters from the immune system. Immunol. Rev..

[37] Anderson K.S., LaBaer J. (2005). The sentinel within: Exploiting the immune system for cancer biomarkers. J. Proteome Res..

[38] Mongre R.K., Sodhi S.S., Sharma N., Ghosh M., Kim J.H., Kim N., Park Y.H., Shin Y.G., Kim S.J., Jiao Z.J. (2016). Epigenetic induction of epithelial to mesenchymal transition by LCN2 mediates metastasis and tumorigenesis, which is abrogated by NF-κB inhibitor BRM270 in a xenograft model of lung adenocarcinoma. Int. J. Oncol..

[39] Wojcik E., Kulpa J.K. (2017). Pro-gastrin-releasing peptide (ProGRP) as a biomarker in small-cell lung cancer diagnosis, monitoring and evaluation of treatment response. Lung Cancer.

[40] Rosiek V., Kogut A., Kos-Kudła B. (2023). Pro-Gastrin-Releasing Peptide as a biomarker in lung neuroendocrine neoplasm. Cancers.

[41] Trivers G.E., De Benedetti V.M., Cawley H.L., Caron G., Harrington A.M., Bennett W.P., Jett J.R., Colby T.V., Tazelaar H., Pairolero P. (1996). Anti-p53 antibodies in sera from patients with chronic obstructive pulmonary disease can predate a diagnosis of cancer. Clin. Cancer Res..

[42] Mathew J., Healey G., Jewell W., Murray A., Chapman C., Peek L., Barnes A., Wood W., Robertson J.F., Boyle P. (2010). Demographics of populations at high risk of lung cancer and results of the Early CDT-Lung test. J. Clin. Oncol..

[43] Heineman D.J., Daniels J.M., Schreurs W.H. (2017). Clinical staging of NSCLC: Current evidence and implications for adjuvant chemotherapy. Ther. Adv. Med. Oncol..

