# Supplementary material for: The Usefulness of the Ratio of Antigen–Autoantibody Immune Complexes to Their Free Antigens in the Diagnosis of Non-Small Cell Lung Cancer
Source: Diagnostics (Basel). 2023 Sep 20;13(18):2999. doi: 10.3390/diagnostics13182999 (PMC10529727; doi:10.3390/diagnostics13182999)
Supplement: Supplementary file 1 [file diagnostics-13-02999-s001.zip › diagnostics-2486022-supplementary.pdf]

Supplement

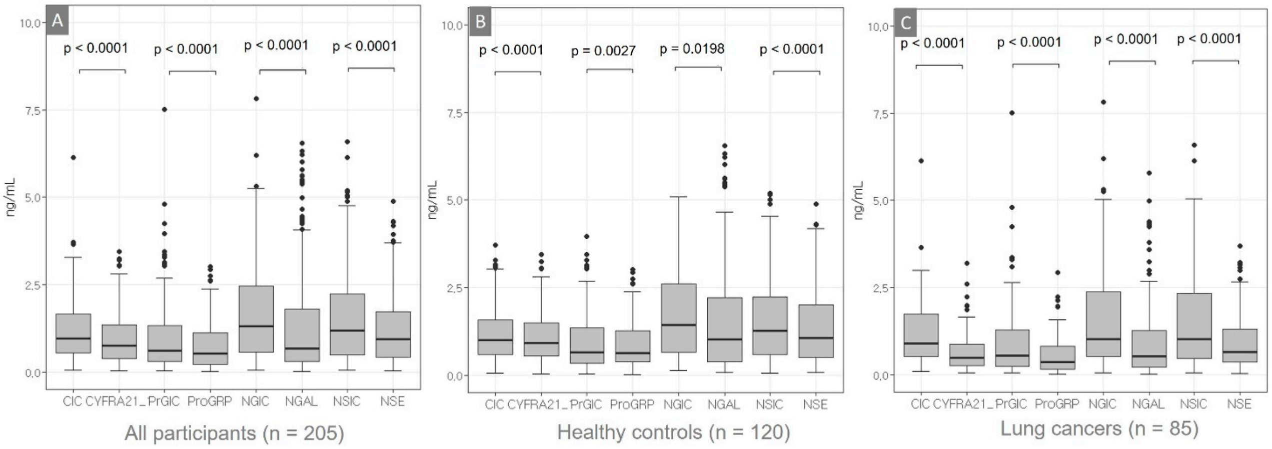

Figure S1. The levels of AICs and their antigens for CYFRA21-1, ProGRP, NGAL, and NSE in all participants (A), in healthy controls (B), and in patients with lung cancers (C). Abbreviations: CIC, CYFRA21-1-Anti-CYFRA21-1 autoantibody immune complex; PrGIC, ProGRP-Anti-ProGRP autoantibody immune complex; NGIC, NGAL-Anti-NGAL autoantibody immune complex; NSIC, NSE-Anti-NSE autoantibody immune complex.

Table S1. The levels of antigen-autoantibody immune complex, and free antigen, their ratios, and combinations from two to four ratios for four proteins.

markers	NSCLC (n = 86)	Healthy Control (n = 120)	p-Value (NSCLC vs. Healthy control)
CIC (median (IQR), ng/mL)	0.91 (0.54, 1.75)	1.00 (0.58, 1.57)	0.7031
CYFRA21-1 (median (IQR), ng/mL)	0.49 (0.26, 0.88)	0.92 (0.56, 1.5)	**<0.0001**
PrGIC (median (IQR), ng/mL)	0.55 (0.24, 1.30)	0.66 (0.34, 1.36)	0.3007
ProGRP, ng/mL (median (IQR), ng/mL)	0.37 (0.16, 0.81)	0.64 (0.39, 1.27)	**0.0002**
NGIC (median (IQR), ng/mL)	1.02 (0.53, 2.38)	1.43 (0.66, 2.61)	0.2680
NGAL (median (IQR), ng/mL)	0.54 (0.22, 1.27)	1.04 (0.38, 2.21)	**0.0015**
NSIC (median (IQR), ng/mL)	1.03 (0.47, 2.34)	1.28 (0.59, 2.23)	0.4056
NSE (median (IQR), ng/mL)	0.65 (0.37, 1.31)	1.07 (0.52, 2.02)	**0.0105**
CIC/ CYFRA21-1 ratio (median (IQR))	1.83 (1.40, 2.50)	1.08 (0.92, 1.3)	**<0.0001**
PrGIC/ ProGRP ratio (median (IQR))	1.69 (1.17, 2.06)	1.06 (0.81, 1.32)	**<0.0001**
NGIC/NGAL ratio (median (IQR))	2.05 (1.39, 2.45)	1.27 (1, 1.65)	**<0.0001**
NSIC/NSE ratio (median (IQR))	1.49 (1.02, 1.82)	1.12 (0.99, 1.39)	**0.0001**
C2-1 (median (IQR))	3.13 (2.19, 4.25)	1.16 (0.81, 1.61)	**<0.0001**
C2-2 (median (IQR))	3.25 (2.29, 5.83)	1.39 (1.06, 1.95)	**<0.0001**
C2-3 (median (IQR))	2.69 (1.85, 4.04)	1.29 (0.95, 1.75)	**<0.0001**
C3-1 (median (IQR))	5.96 (3.48, 9.59)	1.37 (1.01, 2.02)	**<0.0001**
C3-2 (median (IQR))	4.63 (3.22, 8.41)	1.59 (1.2, 2.31)	**<0.0001**
C3-3 (median (IQR))	3.96 (2.28, 7.35)	1.34 (0.86, 2.17)	**<0.0001**
C4-1 (median (IQR))	8.10 (4.38, 14.07)	1.61 (1.08, 2.64)	**<0.0001**

Abbreviations: CIC, CYFRA21-1-Anti-CYFRA21-1 autoantibody immune complex; PrGIC, ProGRP-Anti-ProGRP autoantibody immune complex; NGIC, NGAL-Anti-NGAL autoantibody immune complex; NSIC, NSE-Anti-NSE autoantibody immune complex; NSCLC, non-small cell lung cancer; IQR, interquartile ranges

Table S2. The area under the curve (AUC)

Variable	AUC	95 % CI	P-value
CIC	0.484	0.403 to 0.565	**0.7022**
CYFRA21-1	0.311	0.236 to 0.385	**< 0.0001**
PrGIC	0.458	0.376 to 0.539	**0.3002**
ProGRP	0.347	0.580 to 0.729	**0.0002**
NGIC	0.455	0.373 to 0.536	0.2675
NGAL	0.630	0.565 to 0.7000	0.0015
NSIC	0.534	0.444 to 0.616	0.4049
NSE	0.605	0.520 to 0.688	0.0104
CIC/CYFRA21-1	0.829	0.769 to 0.889	**< 0.0001**
PrGIC/ProGRP	0.768	0.703 to 0.833	**< 0.0001**
NGIC/NGAL	0.774	0.709 to 0.838	**< 0.0001**
NSIC/NSE	0.656	0.574 to 0.738	**0.0001**
C2-1	0.880	0.831 to 0.929	**< 0.0001**
C2-2	0.879	0.832 to 0.927	**< 0.0001**
C2-3	0.816	0.752 to 0.879	**< 0.0001**
C3-1	0.917	0.878 to 0.956	**< 0.0001**
C3-2	0.882	0.832 to 0.931	**< 0.0001**
C3-3	0.855	0.804 to 0.910	**< 0.0001**
C4-1	0.907	0.863 to 0.951	**< 0.0001**

Abbreviations: CIC, CYFRA21-1-Anti-CYFRA21-1 autoantibody immune complex; PrGIC, ProGRP-Anti-ProGRP autoantibody immune complex; NGIC, NGAL-Anti-NGAL autoantibody immune complex; NSIC, NSE-Anti-NSE autoantibody immune complex; CI, confidence interval

Table S3. The diagnostic efficiency of the C4-1 including false positive and false negative rates at the specificity of 86.7% according to stage in patients with NSCLC.

Stage (number)	Sensitivity (95% CI)	PPV (95% CI)	NPV (95% CI)	False Positive (95% CI)	False Negative (95% CI)
CIS (n=2)	100<br>(19.8–100)	11.1<br>(1.9–36.1)	100<br>(95.6–100)	88.9<br>(63.9–98.1)	0<br>(0–4.43)
Stage I (n=39)	89.7<br>(74.8–96.7)	68.6<br>(54.0–80.5)	96.3<br>(90.2–98.8)	31.4<br>(19.5–46.0)	3.7<br>(1.2–9.8)
Stage II (n=16)	62.5<br>(35.9–83.7)	38.5<br>(20.9–59.3)	94.5<br>(88.0–97.8)	61.5<br>(40.7–79.1)	5.5<br>(2.2–12.0)
Stage III (n=24)	91.7<br>(71.5–98.5)	57.9<br>(40.9–73.2)	98.1<br>(92.7–99.7)	42.1<br>(26.7–59.1)	1.9<br>(0.3–7.3)
Stage IV (n=4)	100<br>(39.6–100)	20.0<br>(6.6–44.3)	100<br>(55.7–93.4)	80.0<br>(55.7–93.4)	0<br>(0–4.4)
Very early-stage<br>CIS (0)–I (n=41)	90.2<br>(75.9–96.8)	69.8<br>(55.5–81.3)	96.3<br>(90.2–98.8)	20.2<br>(18.7–44.5)	3.7<br>(1.2–9.8)
Stage II–IV (n=44)	81.8<br>(64.2–89.7)	69.2<br>(54.7–80.9)	92.9<br>(86.0–96.6)	30.8<br>(19.1–45.3)	7.1<br>(3.4–14.0)

Abbreviations: CIS, Carcinoma in situ; PPV, positive predictive value; NPV, negative predictive value; NSCLC, non-small cell lung cancer; CI, confidence interval.

Table S4. The diagnostic efficiency of C4-1 including false positive and false negative rates at the specificity of 86.7% according to pathological diagnosis in patients with NSCLC.

Type (number)	Sensitivity (95% CI)	PPV (95% CI)	NPV (95% CI)	False Positive (95% CI)	False Negative (95% CI)
NSCLC (n=85)	85.9 (76.6–92.5)	82.0 (72.5–89.4)	89.7 (82.6–94.5)	18.0 (10.6–27.5)	10.3 (5.5–7.4)
Adenocarcinoma (n=38, 25/4/9/1*)	89.7 (74.8–96.6)	68.6 (54.0–80.5)	96.3 (90.2–98.8)	31.4 (19.7–46.0)	3.7 (1.2–9.8)
Squamous cell carcinoma (n=38, 14/10/12/2*)	81.6 (65.1–91.7)	66.0 (50.6–78.7)	93.7 (87.0–97.2)	34.0 (12.3–49.4)	6.3 (2.8–13.0)
Other NSCLCs (n=9, 3/2/3/1*)	87.5 (46.7–99.3)	30.4 (14.1–53.0)	99.0 (94.0–100.0)	69.6 (47.0–85.9)	1.0 (0.0–6.0)

* Total number of each pathologic type and the number divided into patients with stages 0-I/II/III/IV.

Abbreviations: CIS, Carcinoma in situ; PPV, positive predictive value; NPV, negative predictive value; NSCLC, non-small cell lung cancer; CI, confidence interval.

Table S5. The diagnostic efficiency of each ratio for the four proteins according to pathological diagnosis in patients with NSCLC.

Variable	Pathological type	Sensitivity (95% CI)	Specificity (95% CI)	PPV (95% CI)	NPV (95% CI)
CIC/CYFRA21-1 (cut-off 1.33)	NSCLC	81.2 (71.8–88.8)	77.5 (69.0–84.6)	71.9 (61.8–80.6)	85.3 (77.3–91.4)
	Adenocarcinoma	78.9 (62.2–89.9)		52.6 (39.1–65.8)	92.1 (84.5–96.3)
	Squamous cell carcinoma	84.2 (68.1–93.4)		54.2 (40.8–67.1)	93.9 (86.8–97.5)
	Others	77.8 (40.2–96.1)		20.6 (9.3–38.4)	97.9 (91.9–99.6)
PrGIC/ProGRP (cut-off 1.41)	NSCLC	67.1 (56.0–76.9)	79.2 (70.8–86.0)	69.5 (58.4–68.8)	77.2 (68.8–84.3)
	Adenocarcinoma	68.4 (51.2–82.0)		51.0 (36.8–65.0)	88.8 (80.9–93.8)
	Squamous cell carcinoma	68.4 (51.2–82.0)		51.0 (36.8–65.0)	88.8 (80.9–93.8)
	Others	55.6 (22.7–84.7)		16.7 (6.30–35.5)	96.0 (89.4–98.7)
NGIC/NGAL (cut-off 1.55)	NSCLC	69.4 (58.5–79.0)	70.8 (61.8–78.8)	62.8 (52.2–72.5)	76.6 (67.6–84.1)
	Adenocarcinoma	78.9 (62.2–89.9)		46.2 (33.9–58.9)	91.4 (83.3–95.9)
	Squamous cell carcinoma	57.9 (40.9–73.3)		38.6 (26.3–52.4)	84.2 (75.2–90.4)
	Others	77.8 (40.2–96.1)		16.7 (7.5–32.0)	97.7 (91.2–99.6)
NSIC/NSE (cut-off 1.22)	NSCLC	63.5 (52.4–73.7)	60.8 (51.5–69.6)	53.5 (43.3–63.5)	70.2 (60.4–78.8)

	Adenocarcinoma	63.2<br>(46.0–77.7)		33.8<br>(23.3–46.1)	83.9<br>(74.1–90.6)
	Squamous cell carcinoma	60.5<br>(43.5–75.5)		32.9<br>(22.4–45.2)	83.0<br>(73.1–89.8)
	Others	77.8<br>(40.2–96.1)		13.0<br>(5.8–25.5)	97.3<br>(89.8–99.5)

Abbreviations: PPV, positive predictive value; NPV, negative predictive value; NSCLC, non-small cell lung cancer; CI, confidence interval.
